# LincRNA Plays a Role in the Effect of *CYP46A1* Polymorphism in Alzheimer’s Disease – Related Pathology

**DOI:** 10.3389/fnagi.2019.00381

**Published:** 2020-01-21

**Authors:** Yang Chen, Hui-Yun Li, Fan Zeng, Le Chen, Fa-Ying Zhou, Ze-Yan Peng, Hai Yang, Hua-Dong Zhou, Yan-Jiang Wang, Ling Li

**Affiliations:** ^1^Department of Neurology, Centre for Clinical Neuroscience, Daping Hospital of Army Medical University, Chongqing, China; ^2^Postgraduate School, Bengbu Medical College, Bengbu, China

**Keywords:** *CYP46A1*, long intronic non-coding RNA, 24-OHC, amyloid β, Alzheimer’s disease

## Abstract

Polymorphism of the cholesterol-24S-hydroxylase (*CYP46A1*) gene is thought to be a risk factor for Alzheimer’s disease (AD). A single nucleotide polymorphism (T/C) in intron 2, rs754203, has been confirmed to be implicated in AD. Rs754203 is located in the long intronic non-coding RNA (LincRNA) sequence, which has previously been shown to be involved in the pathology of many diseases. Thus, the present study aimed to investigate the role of LincRNA in the *CYP46A1* gene expression and related AD pathology. SH-SY5Y cells with overexpressed TT or CC genotype *CYP46A1* were used. Through RT-PCR, Western blot and ELISA assays, we found that LincRNA can affect the *CYP46A1* gene expression and the production of 24-OHC and Aβ. Overexpression of LincRNA can significantly inhibit CYP46A1 expression and 24-OHC production, as well as increasing the Aβ expression level. Silencing of LincRNA confirmed the role that it plays in the regulation of *CYP46A1*, as well as the production of 24-OHC and Aβ. In addition, this effect was stronger in the A type LincRNA than in the G type LincRNA. Results from dual luciferase assays show that LincRNA inhibited the activity of the *CYP46A1* gene promoter. This study indicates a possible novel role of LincRNA and provides a new way to look into the relationship between *CYP46A1* polymorphism and AD pathology. This may identify a novel pathway through which to explore AD therapy.

## Introduction

As the most common form of dementia, Alzheimer’s disease (AD) is affecting an increasing number of people, due to population growth and aging (Hodson, 2018). AD is thought to result from both genetic and environmental factors (Styczynska et al., 2008). *APP*, *PSEN1*, *PSEN2*, and *APOE* genes have all been shown to be related to AD occurrence. Among these genes, *APOE* is the only one that is accepted to be correlated with sporadic AD (St George-Hyslop, 2000). Because APOE is a major cholesterol carrier in the central nervous system, cholesterol metabolism is believed to play a role in AD pathology (Martins et al., 2009). In the adult brain, cholesterol is mainly synthesized by astrocytes. However, cholesterol cannot be cleared in the brain. Instead, cholesterol 24S-hydroxylase (CYP46A1), which is produced by neurons, converts cholesterol into 24S-hydroxycholesterol (24-OHC), which can pass the brain blood barrier and be degraded in the liver (Lutjohann et al., 1996). Plasma 24-OHC is mainly produced by cholesterol metabolism in the brain and can reflect both CYP46A1 activity and the cholesterol level in the brain (Bjorkhem et al., 1998). When the production of cholesterol is increased or its exportation is decreased, it can accumulate in the brain, which can activate β-secretase and γ-secretase, resulting in amyloid β (Aβ) production. Aβ deposition is an important pathological feature of AD (Wahrle et al., 2002; Sidera et al., 2005). As the key regulator of cholesterol metabolism, the *CYP46A1* gene is thought to be related to AD (Garcia et al., 2009). Overexpression of *CYP46A1* can reduce the production and deposition of Aβ in the cortex and thalamus and improve the spatial cognition in a mouse model of AD (Hudry et al., 2010). Hippocampal neuron injury in a stress state occurs more often when *CYP46A1* expression is inhibited (Martin et al., 2011). CYP46A1 may play a role in protecting neurons in the brain and decreased *CYP46A1* expression may be involved in the pathology of AD.

The human *CYP46A1* gene is located at 14q32.1 and consists of 15 exons and 14 introns. Because CYP46A1 is only expressed in the brain, the present studies on the relationship between CYP46A1 and AD were mainly performed on the single nucleotide polymorphisms (SNP) linked to AD susceptibility. Many *CYP46A1* SNP sites have been discovered but rs754203 is the only one that has been reported to be related to AD occurrence (Johansson et al., 2004; Fernandez Del Pozo et al., 2006; Garcia et al., 2009). Rs754203 is located at the second intron in the *CYP46A1* gene and shows T→C polymorphism. Although there have been contradictory reports on the role of *CYP46A1* polymorphism in AD (Papassotiropoulos et al., 2003; Borroni et al., 2004; Helisalmi et al., 2006; Li et al., 2006; Tedde et al., 2006; Kolsch et al., 2009), it has been reported that the *CYP46A1* T allele may be a risk factor (He et al., 2012). Furthermore, AD patients with the TT *CYP46A1* genotype have higher 24-OHC levels than those observed in others (Li et al., 2018). Overall, these studies have confirmed a correlation between *CYP46A1* polymorphism and AD. However, the detailed mechanisms require further investigation.

**FIGURE 1:**
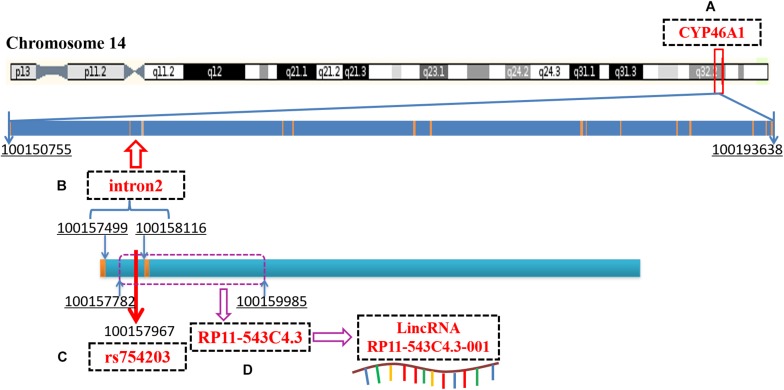
A diagram of *CYP46A1* gene, rs754203 locus and RP11-543C4.3-001 LincRNA transcription sequence. **(A)** The human *CYP46A1* gene is located at 14q32.1 and consists of 15 exons and 14 introns, with a total length of 42,884bp. **(B)** The second intron is 618bp long and locates on chromosome 14, 100157499-100158116. **(C)** Rs754203 is located at 469 base of the second intron (100157967). **(D)** The transcription sequence of RP11-543c4.3-001 LincRNA, RP11-543C4.3, is located on chromosome 14, 100157782-100159985.

Recent reports have demonstrated that introns can regulate gene expression by interfering with mRNA splicing, maturation or through non-coding RNA (Chernikova et al., 2016; Kumari et al., 2018; Pieczynski et al., 2018; Thakran et al., 2018). Intron 2 of *CYP46A1*, which is the location of the SNP site, rs754203, contains the sequence of long intronic non-coding RNA (LincRNA), RP11-543C4.3-001 (Figure 1). The LincRNA has been reported to be the potential target of some diseases. LincRNA-EPS was found to be a repressor of inflammatory responses and to play an important role in the immune system (Atianand et al., 2016). The haplotype of *LincRNA-21* was shown to be associated with a decreased risk of coronary artery disease and myocardial infarction (Tang et al., 2016). LincRNA is involved in the pathogenesis of many diseases mainly by regulating the expression of its mother gene. The detailed mechanisms through which it acts include the binding of promoters or transcription factors, influencing the alternative splicing of mRNA, and influencing the stability of mRNA. Thus, LincRNA RP11-543C4.3-001 may also play a role in the regulation of *CYP46A1* expression. Investigation into the effect of RP11-543C4.3-001 on *CYP46A1* expression, as well as its role in cholesterol metabolism and Aβ production, may provide more evidence and reveal a new target for the treatment of AD pathology.

Therefore, in the present study, we adopted an *in vitro* cell model with the *CYP46A1* TT or CC genotype, and investigated the role of LincRNA RP11-543C4.3-001 in the effect of *CYP46A1* polymorphism in AD-related pathological features, such as 24-OHC and Aβ production, to clarify its role in AD pathology.

## Materials and Methods

### Plasmid Construction

Total RNA was extracted from SH-SY5Y cells with TRIzol reagent (Beyotime, China) and transferred to total cDNA through reverse transcription with the PrimeScript RT reagent Kit (Takara, Japan). *CYP46A1* cDNA was amplified by RT-PCR and sequenced to obtain CC and TT genotypes of *CYP46A1* (Western Biotechnology, China). Genome DNA was obtained from SH-SY5Y cells using the genome DNA extraction kit (Qiagen, Germany). TT and CC genotype *CYP46A1* promoters and the corresponding transcriptional sequences of A and G type LincRNA RP11-543c4.3-001 were cloned from the genome DNA.

**FIGURE 2:**
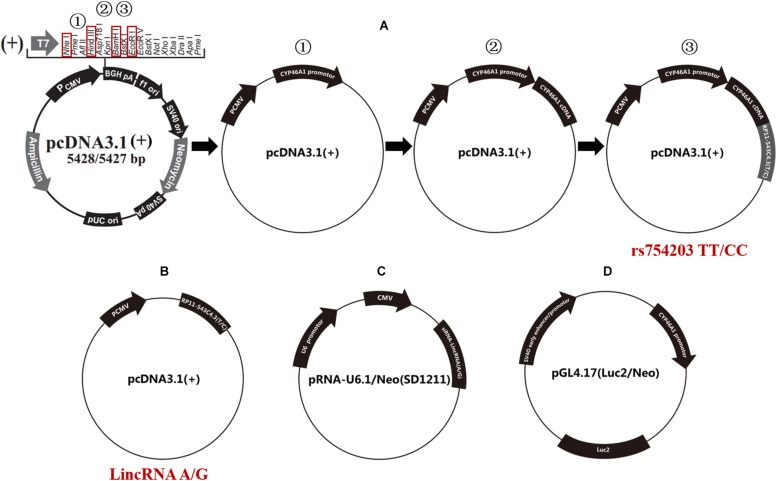
Schematic diagram of the plasmids that were used in the current study. **(A)** The procedure for the construction of the rs754203 TT and CC genotype *CYP46A1* containing plasmid. **(B)** The plasmid containing LincRNA RP11-543C4.3-001 A/G transcription sequence. **(C)** The plasmid containing LincRNA A/G siRNA transcription sequence. **(D)** The luciferase report gene plasmid containing *CYP46A1* promoter sequence.

The human *CYP46A1* promoter, rs754203 T/C RP11543c4.3 (A/G type LincRNA) transcription sequences and *CYP46A1* cDNA were synthesized and cloned into pcDNA3.1 using three rounds of double endonuclease reactions (Western Biotechnology, China). The following enzymes were used: *Nhe*I and *Hin*dIII, *Eco*RI and *Hin*dIII, and *Bam*HI and *Hin*dIII (Takara, Japan). Finally, plasmid pcDNA3.1-CYP46A1promoter-RP11-543C4.3-CYP46A1 cDNA were constructed and confirmed by sequencing (rs754203 TT/CC, Figure 2A).

Furthermore, A/G type LincRNA RP11543c4.3-001 plasmids were constructed based on the pcDNA3.1 for LincRNA overexpression experiments [LincRNA(A/G), Figure 2B] using the following enzymes: *Nhe*I and *Hin*dIII. LincRNA(A/G) siRNA-LincRNA(A/G) was synthesized by Western Biotechnology, China. siRNA plasmids were constructed based on the pRNA-U6.1/Neo (SD1211) for siRNA silencing trials (Figure 2C), using the following enzymes: *Bam*HI and *Hin*dIII. The luciferase reporter gene pGL4.17-*CYP46A1* was also constructed to explore the function of the *CYP46A1* promoter (Figure 2D), using the following enzymes: *Xho*I and *Hin*dIII.

### SH-SY5Y Cell Culture and Transfection

The human neuroblastoma cell line SH-SY5Y cells (National Infrastructure of Cell Line Resource, China) were cultured in Dulbecco’s modified Eagle medium/nutrient mixture F-12 medium (Gibco, United States) supplemented with 10% fetal bovine serum, 100 IU/ml penicillin and 100 μg/ml streptomycin at 37°C in a humidified 5% CO_2_ incubator (Li et al., 1996). Cells were grown to 70–80% confluence in 25 mm diameter dishes and fed every fourth day.

Transfection was performed using the Lipofectamine 2000 reagent (Invitrogen, United States) Plasmids (mentioned above) were transfected into cells 24 h before subsequent treatments. Blank plasmid was used as the control condition. Cell models were confirmed by RT-PCR. Following transfection, 10 μmol/L cholesterol (Sigma, United States) was added to the cell medium for 48 h.

### Detection of the Expression of Long Intronic Non-coding RNA and *CYP46A1*

After treatments, SH-SY5Y cells were harvested. The total RNA was extracted using TRIzol reagent and transferred to total cDNA using reverse transcription. The contents of LincRNA RP11-543C4.3-001 and *CYP46A1* mRNA were measured through real-time PCR (Takara, Japan). The primers used for RP11-543C4.3-001 LincRNA were: RP11-543C4.3-001F TGCTACCAAAAGAGTGCTGTCC; RP11-543C4.3-001R GAG TGTTTCCAACCCTATTCCA. The primers used for CYP46A1 were: hCYP46A1F CGCTACGAGCACATCCCC; hCYP46A1R CCCGCACAACAGGTCCATAC.

CYP46A1 protein levels were detected by Western blot. Briefly, SH-SY5Y cells were harvested in ice-cold RIPA buffer (Beyotime, China) containing 1 mM PMSF. The samples were then centrifuged at 13500 *g* at 4°C for 15 min and the supernatants were collected. The protein content was measured using the bicinchoninate acid (BCA) assay kit (Takara, Japan). Samples were stored at −80°C until further use. For immunoblotting analysis, protein samples of brain tissues and cell lysate were mixed with 5 × SDSPAGE Sample Buffer and boiled for 10 min. Aliquots of cell lysate (∼20 μg) were separated on a 12% SDSPAGE and then transferred onto PVDF membranes (Millipore, United States). The non-specific binding sites of the membranes were blocked with 5% non-fat milk in phosphate buffered saline containing 0.1% Tween-20 (PBST, pH 7.4) for 1 h at room temperature. The membranes were then incubated overnight at 4°C with rabbit anti CYP46A1 antibody (Abcam, United States). The following day, the membranes were washed three times and then incubated for 1 h at room temperature with a horseradish peroxidase-linked secondary anti-rabbit antibody (1:5000 dilution; Sigma, United States). The membranes were washed again, three times with PBST, and the immunoreactive bands were then visualized using Super ECL plus chemiluminescence detection reagents (Thermo Fisher Scientific, United States). The immunoreactive bands were visualized and quantified by Bio-RAP (Bio-rad, United States). GAPDH (Abcam, United States) was used as a loading control.

### Measurement of Aβ and 24-OHC Content

Aβ1–40, Aβ1–42 and plasma 24-OHC levels were measured using an enzyme-linked immunosorbent assay (ELISA) kit (Beijing Like Biotechnology, China) according to the manufacturer’s instructions. Samples and standards were measured in duplicate, and the mean of the duplicates was used for statistical analyses.

### Dual Luciferase Assays

Dual luciferase assays were performed by using the dual luciferase reporter assay system (Promega, United States) according to the manufacturer’s instructions. After co-transfection of pGL4.17-*CYP46A1* and pcDNA3.1(+)-RP11-543C4.3(T/C) for 48 h, 150 μl of cell lysate was taken and transferred to 75 μl of 1 × passive lysis buffer. After lysis for 10 to 15 s, a 10-μl aliquot was used for luminescence measurements with a microplate reader (Thermo Fisher Scientific, United States).

### Statistical Analysis

All reported values represent means ± standard deviation (SD). Data were analyzed using SPSS 18.0, GraphPad Prism 5, and Microsoft Excel 2016 statistical software. Differences between experimental groups were considered significant at a probability <0.05 on a two-tailed test; ^∗^*P* < 0.05, ^∗∗^*P* < 0.01, and ^∗∗∗^*P* < 0.001 vs. control, respectively, #*P* ≥ 0.05.

## Results

### Long Intronic Non-coding RNA Can Affect the Expression of *CYP46A1*

**FIGURE 3:**
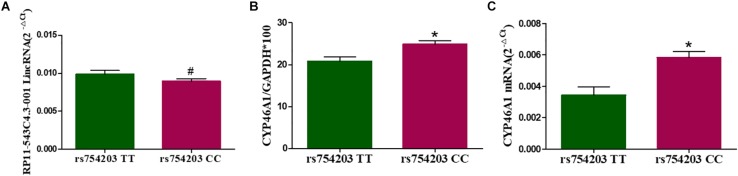
The expression of LincRNA and *CYP46A1* in SH-SY5Y cells transfected with the rs754203 TT and CC genotype of *CYP46A1*. Plasmids containing the rs754203 TT or CC genotype of *CYP46A1* were transfected into SH-SY5Y cells. After 24 h, cells were harvested for RT-PCR and Western blot. The *CYP46A1* mRNA level **(A)**, CYP46A1 protein level **(B)**, and LincRNA RP11543c4.3-001 level **(C)** are shown. *p < 0.05 and #*p* ≥ 0.05.

To investigate whether LincRNA can influence the expression of the *CYP46A1* gene, whose polymorphism is thought to be involved in AD, we firstly constructed cell models of the different *CYP46A1* genotypes *in vitro* through the transfection of SH-SY5Y cells. Plasmids bearing *CYP46A1* rs754203 TT/CC were transfected into SH-SY5Y cells and cultured cells for 24 h. Cells were harvested for the measurement of *CYP46A1* content and LincRNA A/G RP11543c4.3-001. As shown, after transfection, *CYP46A1* expression differed between cells that were transfected with the rs754203 TT and CC genotype, at both the mRNA and protein level. CC genotype transfected cells contained a higher level of *CYP46A1* mRNA and protein than TT genotype transfected cells (Figures 3A,B). Meanwhile, no difference was observed in the LincRNA level between the cells transfected with the rs754203 CC and TT genotype *CYP46A1* (Figure 3C).

**FIGURE 4:**
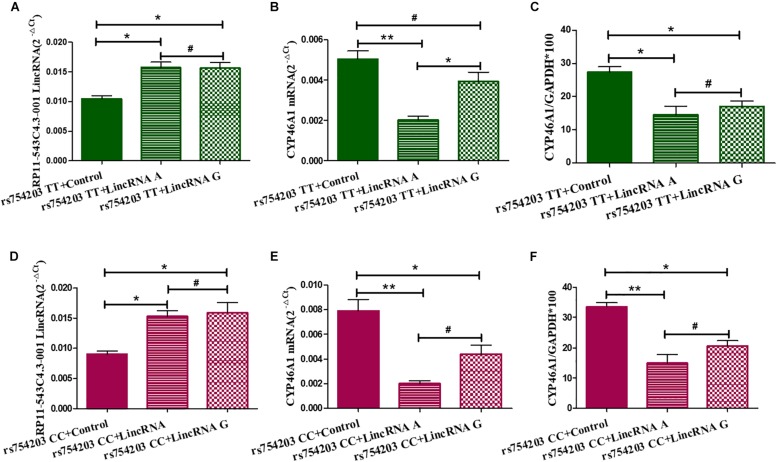
Changes in the expression of LincRNA and *CYP46A1* after LincRNA overexpression in rs754203 TT and CC genotype *CYP46A1* transfected SH-SY5Y cells. Plasmids containing rs754203 TT or CC genotype of *CYP46A1* were transfected into SH-SY5Y cells. After 24 h, LincRNA A/G was transfected into cells for 48 h. Through RT-PCR and Western blot, the LincRNA RP11543c4.3-001 level **(A,B)**, *CYP46A1* mRNA level **(C,D)**, and CYP46A1 protein level **(E,F)** were measured. *p < 0.05, **p < 0.01, and #*p* ≥ 0.05.

Next, we overexpressed different types of LincRNA RP11543C4.3-001 (A/G) in the TT or CC genotype *CYP46A1* cells, since LincRNA usually regulates the expression of the gene that it comes from. We wanted to investigate whether the change in the expression of LincRNA RP11543C4.3-001 could affect the *CYP46A1* expression. As shown, LincRNA RP11543C4.3-001 levels were higher in LincRNA transfected cells than in non-transfected cells in both the rs754203 TT and CC genotype conditions (Figures 4A,B). No significant differences were observed between the LincRNA A condition and LincRNA G conditions. As shown in Figures 4C–F, both the A and G type LincRNA can induce a lower level of *CYP46A1* mRNA and protein expression in both the rs754203 TT and CC genotype conditions. By comparison, the inhibitory effect of LincRNA A was more significant than that of LincRNA G. Thus, we concluded that LincRNA RP11543C4.3-001 can induce a decrease in the *CYP46A1* expression.

**FIGURE 5:**
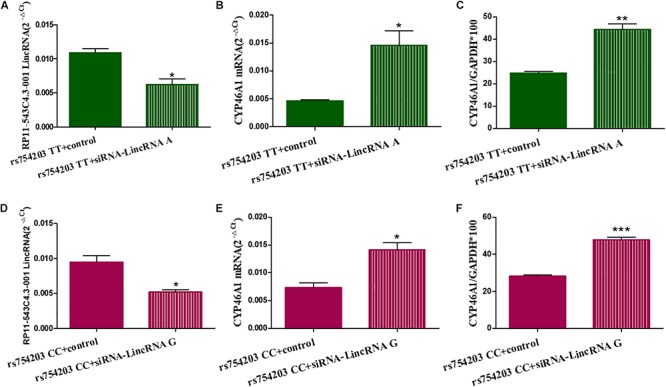
Changes in the expressions of LincRNA and *CYP46A1* after LincRNA silencing in the rs754203 TT and CC genotype of *CYP46A1* transfected SH-SY5Y cells. Plasmids containing the rs754203 TT or CC genotype of *CYP46A1* were transfected into SH-SY5Y cells. After 24 h, LincRNA siRNA were transfected into the cells for 48 h. Through RT-PCR and Western blot, the LincRNA RP11543c4.3-001 level **(A,B)**, *CYP46A1* mRNA level **(C,D)**, and CYP46A1 protein level **(E,F)** were measured. *p < 0.05, **p < 0.01, and ∗∗∗*p* < 0.001.

To confirm the effect of LincRNA RP11543C4.3-001 in the downregulation of *CYP46A1* expression, we performed siRNA silencing experiments using the rs754203 TT and CC genotype *CYP46A1* cell models. Firstly, we observed that the level of LincRNA RP11543C4.3-001 was significantly reduced after silencing (Figures 5A,B). After the knockdown of LincRNA RP11543C4.3-001, *CYP46A1* mRNA and protein levels were shown to be significantly higher in both the rs754203 TT genotype and CC genotype conditions than the levels observed in the control (Figures 5C–F). In summary, the above data indicates that LincRNA RP11543C4.3-001 can reduce *CYP46A1* expression, at both the mRNA and protein levels.

### Long Intronic Non-coding RNA Suppresses the Activity of the *CYP46A1* Gene Promoter

**FIGURE 6:**
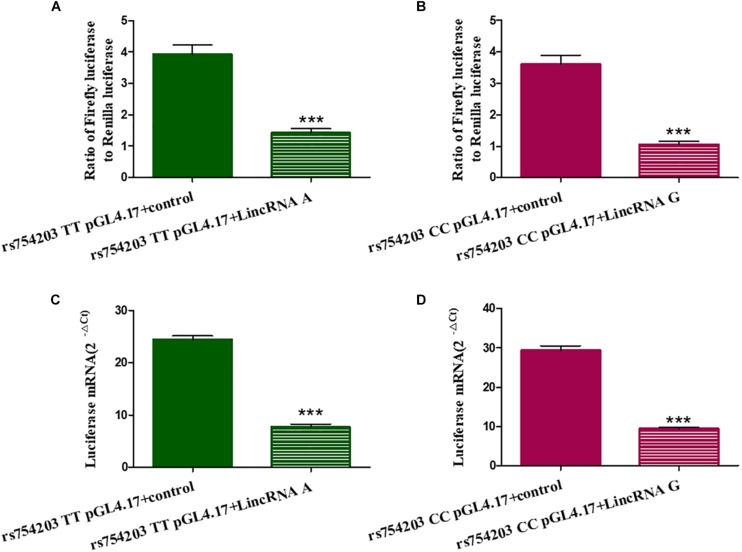
Influence of LincRNA on the activity of the *CYP46A1* gene promoter. Plasmids containing *CYP46A1* rs754203 TT/CC and LincRNA RP11543C4.3 A/G were co-transfected into SH-SY5Y cells. Dual luciferase assays were performed. The ratio of Firefly luciferase to Renilla luciferase **(A,B)** and luciferase mRNA level **(C,D)** are shown. ∗∗∗*p* < 0.001.

To further investigate the mechanism through which LincRNA regulates *CYP46A1* expression, we adopted dual luciferase assays to observe whether LincRNA RP11543C4.3-001 can interfere with the activity of the *CYP46A1* gene promoter. The results show that both LincRNA A and LincRNA G decreased the expression of luciferin. This indicates that there is an inhibitory effect of LincRNA RP11543C4.3-001 on the activity of the gene promoter (Figure 6). However, there was no significant difference between the LincRNA A and G conditions (*p* = 0.920 for Fluc/Rluc and *p* = 0.109 for Luciferase mRNA level comparisons). The change in the *CYP46A1* expression level following transfection with LincRNA A/G may not result from their influence on the gene promoter.

### Long Intronic Non-coding RNA Affects 24-OHC and Aβ Production in SH-SY5Y Cells

**FIGURE 7:**
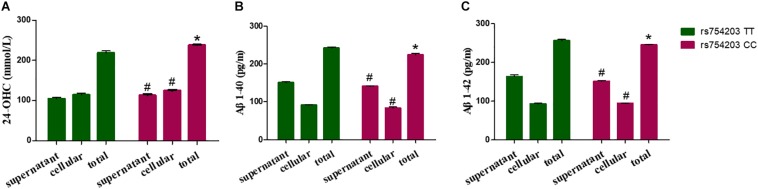
The production of 24-OHC and Aβ in SH-SY5Y cells transfected with the rs754203 TT and CC genotype of *CYP46A1*. Plasmids containing the rs754203 TT or CC genotype of *CYP46A1* were transfected into SH-SY5Y cells. After 24 h, cells were harvested for ELISA assays. 24-OHC **(A)**, Aβ1–40 **(B)**, and Aβ1–42 **(C)** levels are shown. *p < 0.05 and #*p* ≥ 0.05.

Because LincRNA can affect *CYP46A1* expression, which is important in AD pathology, we wanted to investigate whether LincRNA can influence the production of some key AD-related markers, such as 24-OHC and Aβ. To this end, we also used the *CYP46A1* transfected SH-SY5Y cell model. After the SH-SY5Y cells were transfected with *CYP46A1* for 24 h, cholesterol was added to the cell culture medium to measure the level of cholesterol metabolism in neuronal cells. We measured the 24-OHC level in the cells and the medium. The results demonstrate that the total level of 24-OHC was higher in the rs754203 CC genotype condition than in the rs754203 TT genotype condition (Figure 7A). We also measured the level of Aβ production. Both Aβ1–40 and Aβ1–42 production were lower in the rs754203 CC genotype condition than in the rs754203 TT genotype condition (Figures 7B,C). These results suggest that *CYP46A1* polymorphism can alter cholesterol metabolism and Aβ production.

**FIGURE 8:**
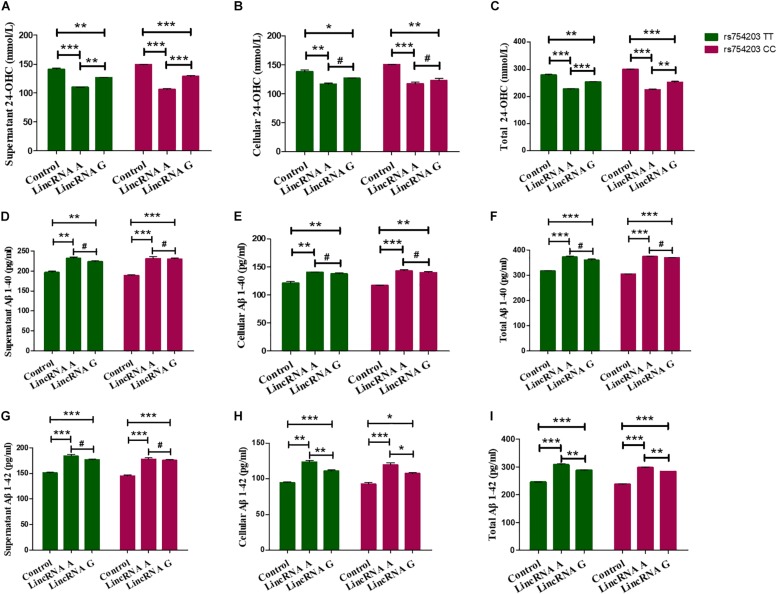
Changes in the production of 24-OHC and Aβ after LincRNA overexpression in rs754203 TT and CC genotype *CYP46A1* transfected SH-SY5Y cells. Plasmids containing rs754203 TT or CC genotype of *CYP46A1* were transfected into SH-SY5Y cells. After 24 h, LincRNA A/G was transfected into cells for 48 h. Through ELISA assays, 24-OHC **(A–C)**, Aβ1–40 **(D–F)**, and Aβ1–42 **(G–I)** were measured. *p < 0.05, **p < 0.01, ∗∗∗*p* < 0.001, and #*p* ≥ 0.05.

Next, 24-OHC and Aβ production were measured following LincRNA RP11543C4.3-001 overexpression. Similar to the changes in the levels of CYP46A1 observed, 24-OHC production in both the cells and the medium were lower in LincRNA RP11543C4.3-001 transfected cells than in non-transfected cells (Figures 8A–C). Additionally, the reduction in 24-OHC production was greater in the LincRNA A condition than in the LincRNA G condition, suggesting that the inhibitory effect was stronger in this condition. The Aβ production was higher in both the cells and medium in the LincRNA RP11543C4.3-001 overexpression condition compared to the control (Figures 8D–I). The increase in Aβ1–42 production was also higher in the LincRNA A condition than in the LincRNA G condition. However, there was no significant difference in the Aβ1–40 production between the two types of LincRNA. These data indicate that LincRNA RP11543C4.3-001 can affect 24-OHC and Aβ production in our cell models. LincRNA RP11543C4.3-001 induces a reduced level of 24-OHC production, and an increase in Aβ production. Meanwhile, there was a significant difference in the effect of the different types of LincRNA, which result from different genotypes of the *CYP46A1* gene. The effect of LincRNA A was more significant than that of LincRNA G. This suggests that there is a high risk of observing the rs754203 T allele in the *CYP46A1* gene in AD.

**FIGURE 9:**
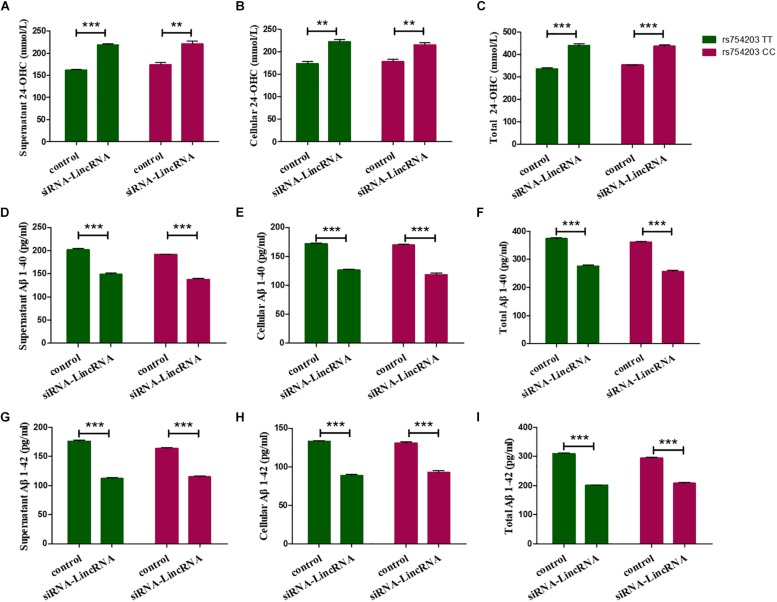
Changes in the productions of 24-OHC and Aβ after LincRNA silencing in the rs754203 TT and CC genotype of *CYP46A1* transfected SH-SY5Y cells. Plasmids containing the rs754203 TT or CC genotype of *CYP46A1* were transfected into SH-SY5Y cells. After 24 h, LincRNA siRNA were transfected into the cells for 48 h. Through ELISA assays, 24-OHC **(A–C)**, Aβ1–40 **(D–F)**, and Aβ1–42 **(G–I**) were measured. **p < 0.01 and ∗∗∗*p* < 0.001.

Furthermore, 24-OHC and Aβ production were measured following the silencing of LincRNA RP11543C4.3-001. Silencing of LincRNA RP11543C4.3-001 resulted in an increase in the production of 24-OHC, both inside the cells and in the medium (Figures 9A–C). The Aβ production, of both Aβ1–40 and Aβ1–42, was significantly reduced after the silencing of LincRNA RP11543C4.3-001 (Figures 9D–I). The level of Aβ1–40 and Aβ1–42 that were detected in both the cell pellet and culture medium were reduced following the inhibition of LincRNA expression.

Combined with the results above, our work demonstrates that LincRNA can inhibit *CYP46A1* expression, suppress the activity of *CYP46A1* gene promoter and alter 24-OHC and Aβ production. The Rs754203 T allele of the *CYP46A1* gene and the resultant A type LincRNA had a more harmful effect on the cultured SH-SY5Y cells. This work has the potential to give rise to a new direction for the investigation of LincRNA and AD etiology.

## Discussion

The present study aimed to investigate the possible role of LincRNA in the effect of *CYP46A1* polymorphism in AD-like pathology using *in vitro* cultured SH-SY5Y cells. We constructed rs754203 TT and CC genotype *CYP46A1* overexpression cell models. We found that LincRNA RP11543C4.3-001 can alter the *CYP46A1* expression. LincRNA RP11543C4.3-001 induced a reduction in the *CYP46A1* expression, at both the mRNA and protein levels. LincRNA RP11543C4.3-001 also decreased 24-OHC production and increased Aβ production inside cells and in the secreted medium. The effects of the A type LincRNA on the inhibition of *CYP46A1* expression, the decrease in 24-OHC production and the increase in Aβ production were stronger than those of the G type LincRNA. Furthermore, dual luciferase assays indicated that LincRNA RP11543C4.3-001 can suppress the activity of the *CYP46A1* gene promoter. As far as we know, this is the first report to provide evidence that LincRNA regulated gene expression can influence the resultant AD-linked pathology (24-OHC and Aβ). This finding may provide more evidence for the involvement of cholesterol metabolism in AD pathology and provide a new target for the treatment of the disease.

Along with the *APOE* gene, the *CYP46A1* gene is thought to be related to AD susceptibility (Corder et al., 1993; Bertram et al., 2007). As a cytochrome P450 family enzyme, CYP46A1 converts cholesterol to 24-OHC in the brain, enabling its entrance into the circulation and its degradation. This is the only way that cholesterol in the brain is metabolized, making CYP46A1 important for the maintenance of cholesterol homeostasis in the central nervous system. *CYP46A1* gene polymorphisms have been reported to be involved in the pathogenesis of AD. However, this topic is controversial. The majority of the studies on this topic have been based on association analysis of the occurrence of the *CYP46A1* genotype in the AD and control population. Further biomedical and mechanism-focused investigations should be performed to clarify the role of CYP46A1 in AD. In our previous work, we investigated the polymorphisms of *CYP46A1*, along with the blood concentrations of 24-OHC and Aβ in patients with AD in a large population (Li et al., 2018). We found that the *CYP46A1* C allele was linked to a higher risk of AD through regression analysis. The 24-OHC and Aβ levels were higher in patients with AD than in control participants. However, patients with AD with the *CYP46A1* TT genotype, but not the CC genotype, showed higher levels of 24-OHC. This suggests that other mechanisms may be involved in the relationship between *CYP46A*1 polymorphisms and AD (Li et al., 2018). In the present study, we used an *in vitro* cell model to clarify the possible role of LincRNA in the effect of *CYP46A1* polymorphism in AD-like pathology. The results show that the TT genotype of *CYP46A1* resulted in reduced *CYP46A1* expression and 24-OHC production, along with an increase in Aβ production. These data suggest that the TT genotype of *CYP46A1* might be the risk genotype of AD. This study solely used an *in vitro* model and, thus, the findings need to be confirmed *in vivo*. This finding opposes the previous finding that patients with AD with the TT genotype of *CYP46A1* have higher levels of 24-OHC than controls. However, considering the different system used in these experiments (*in vitro* cell model and humans) and the complexity of the human body, it is hard to reach a conclusion about which genotype of *CYP46A1* is more harmful. Much more investigation, both association analysis and experimental laboratory investigations are needed to provide further clarification.

The most important finding of the present study is that LincRNA appears to be involved in the relationship of *CYP46A1* polymorphism and AD pathology. As shown, TT genotype, but not CC genotype, *CYP46A1* cells showed higher Aβ and lower 24-OHC expression levels than those observed in control conditions. Because LincRNA RP11543C4.3-001 shares the same intronic location with TT polymorphic loci of *CYP46A1* gene, there is a chance that it could be an antisense to *CYP46A1* gene. Further investigations should be carried to clarify this. Overexpression of LincRNA RP11543C4.3-001 in *CYP46A1* cells can decrease *CYP46A1* expression, increase Aβ1–40 and Aβ 1-42 levels, and decrease 24-OHC levels. Silencing of LincRNA RP11543C4.3-001, using siRNA, can rescue these effects significantly, resulting in a higher level of *CYP46A1* expression, decreased Aβ production and increased 24-OHC production. These data indicate that LincRNA can regulate *CYP46A1* expression and influence 24-OHC and Aβ production in these cells. This effect may result from the inhibition of the promoter activity of the *CYP46A*1 gene, as indicated by the dual luciferase assay results. This may be a novel mechanism through which *CYP46A1* polymorphism works in AD pathology. Thus, it would be worthwhile to investigate the effect of LincRNA in animal models and in the human body. This might also be a new path through which to explore cholesterol metabolism in the AD etiology. Further investigations need to be performed to clarify the role of LincRNA in AD pathology.

We also found that the regulation of *CYP46A1* expression, 24-OHC production and Aβ production in SH-SY5Y cells differed between the LincRNA A and G conditions. LincRNA A exhibited significantly stronger effects than LincRNA G. Overexpression of LincRNA A resulted in a reduction in CYP46A1 expression, decreased 24-OHC production and increased Aβ production. Although LincRNA A and G both inhibited the activity of the *CYP46A1* gene promoter, there was no significant difference between the promoter activity in these conditions. This suggests that the difference in the expression level of *CYP46A1* observed in LincRNA A and G conditions did not result from the regulation of the gene promoter activity. The difference may, instead, result from changes in other transcriptional factors involved in the regulation of *CYP46A1* expression, or a difference in the interfering *CYP46A1* mRNA splicing procedure. Several nuclear proteins may mediate the regulation of lincRNA RP11543C4.3-001 on *CYP46A1* gene promoter. To get a preliminary understanding of the relationship between LincRNA RP11543C4.3-001 and *CYP46A1* gene promoter activity, we have run a prediction analysis from public databases. The results showed LincRNA RP11-543C4 may bind to the enhancer of zeste 2 polycomb repressive complex 2 (EZH2)^1^ (Lu et al., 2013), a histone methyltransferase, which could methylate Lys-27 on histone 3 (H3K27me). Further results showed that H3K27Me3 were found in CHIP-Seq results from different cell lines and could inhibit the activity of the *CYP46A1* gene promoter^2^,^3^.

## Conclusion

In conclusion, the present study suggests that LincRNA may play a role in the relationship between *CYP46A1* polymorphism and AD pathology. LincRNA can affect *CYP46A1* expression and 24-OHC and Aβ production, which might provide a novel pathway for the treatment of AD pathology.

## Data Availability Statement

The datasets for this article are not publicly available. Requests to access the datasets should be directed to LL, mailto:connie1230@foxmail.comconnie1230@foxmail.com.

## Author Contributions

LL, Y-JW, and H-DZ conceived and designed the experiments. LC, F-YZ, Z-YP, and HY performed the experiments. LL and FZ analyzed the data. LL, H-DZ, and FZ contributed reagents, materials, and analysis tools. YC and H-YL wrote the manuscript.

## Conflict of Interest

The authors declare that the research was conducted in the absence of any commercial or financial relationships that could be construed as a potential conflict of interest.

## Footnotes

1 https://asp.unmc.edu/AP/main.php

2 http://genome.ucsc.edu

3 http://genome-asia.ucsc.edu
